# Capric Acid Inhibits NO Production and STAT3 Activation during LPS-Induced Osteoclastogenesis

**DOI:** 10.1371/journal.pone.0027739

**Published:** 2011-11-16

**Authors:** Eun-Jung Park, Sun A. Kim, Yong-Min Choi, Hyuk-Kwon Kwon, Wooyoung Shim, Gwang Lee, Sangdun Choi

**Affiliations:** Department of Molecular Science and Technology, Ajou University, Suwon, Korea; Seoul National University College of Medicine, Korea

## Abstract

Capric acid is a second medium-chain fatty acid, and recent studies have shown that fatty acids are associated with bone density and reduce bone turnover. In this study, we investigated the effects of capric acid on lipopolysaccharide (LPS)-induced osteoclastogenesis in RAW264.7 cells. After treatment with capric acid (1 mM), the number of tartrate resistant acid phosphatase (TRAP)-positive cells decreased significantly. Capric acid reduced LPS-induced TRAP expression, an osteoclast differentiation marker, without inhibiting cell viability. LPS strongly upregulated inducible nitric oxide synthase (iNOS) mRNA levels and nitric oxide (NO) production, whereas capric acid inhibited them. Furthermore, capric acid also inhibited monocyte chemoattractant protein-1 (MCP-1) mRNA expression. Subsequently, we investigated various intracellular signaling proteins, including nuclear factor-κB (NF-κB), c-Jun-N-terminal kinase (JNK), extracellular signal regulated kinase 1/2 (ERK1/2), and signal transducer and activator of transcription 1 (STAT1) and STAT3 associated with osteoclastogenesis. Capric acid had no effects on LPS-induced activation of the NF-κB, JNK, ERK1/2, and STAT1 pathways. However, capric acid inhibited LPS-induced phosphorylation of Ser^727^ in STAT3. Additionally, stattic (a STAT3 inhibitor) inhibited LPS-induced iNOS and MCP-1 gene expression. In conclusion, we demonstrated that capric acid inhibited LPS-induced osteoclastogenesis by suppressing NO production via the STAT3 pathway. These results suggest that capric acid has important therapeutic implications for treating bone diseases associated with excessive osteoclastogenesis.

## Introduction

Osteoclasts are mainly derived from bone marrow hematopoietic monocyte/macrophage lineages. Osteoclasts are formed through multiple steps, including cell-to-cell contact, fusion, and differentiation [1]. Osteoclasts are characterized by high expression of tartrate resistant acid phosphatase (TRAP), which can be used as a cytochemical marker for osteoclasts and their precursors. Increased osteoclast activity leads to bone loss and eventually to bone diseases such as induced rheumatoid arthritis and osteoporosis [2], [3], [4], [5], [6]. Therefore, regulation of osteoclastogenesis plays a key role in bone homeostasis.

Cell fusion plays a critical role controlling osteoclastogenesis. Nitric oxide (NO) and monocyte chemoattractant protein-1 (MCP-1) enhance osteoclastogenesis by mediating cell fusion. NO also increases osteoclast formation by increasing actin remodeling in mononuclear pre-osteoclasts, thereby mediating fusion and formation of multinucleated osteoclasts [7]. NO is a short-lived free radical involved in the regulation of many physiological processes such as vascular relaxation, neurotransmission, platelet aggregation, and the immune response [8], [9]. Furthermore, it is generated from oxygen and L-arginine by nitric oxide synthase (NOS). Three isoforms of NOS have been identified: a neuronal form (nNOS or NOS1), an endothelial form (eNOS or NOS3), and an inducible form (iNOS or NOS2). Among these three NOS, iNOS is expressed in response to various inflammatory stimuli and results in the production of a large amount of NO by macrophages during inflammation [10], [11]. Receptor activator of nuclear factor-κB ligand (RANKL) and macrophage colony-stimulating factor are both considered necessary and sufficient for osteoclast formation [1]. Recent studies have reported that lipopolysaccharide (LPS) induces osteoclast differentiation and increases bone loss [12].

LPS, a bacteria-derived cell wall product, has long been recognized as a key factor in the development of bone loss [13]. LPS plays an important role in bone resorption, which involves recruitment of inflammatory cells, synthesis of cytokines [such as interleukin-6 (IL-6), and tumor necrosis factor-α (TNF-α)], and activation of osteoclast formation and differentiation [12]. LPS induces the production of pro-inflammatory mediator by osteoclasts via the nuclear factor-κB (NF-κB) pathway and the three major mitogen-activated protein kinases (MAPKs), extracellular signal regulated kinase 1/2 (ERK1/2), c-Jun-N-terminal kinase (JNK), and p38 [14], [15], [16].

Another important transcription factor involved in inflammatory cytokine induction is signal transducer and activator of transcription (STAT). The STAT family participates in the regulation of genes involved in the acute phase of the inflammatory response, cell growth, and cell differentiation [17]. The ability of STAT family proteins to homo or heterodimerize alters gene transcription based upon their binding to specific response elements in the promoters of target genes [18], [19], [20]. In previous studies, it was shown that the promoter region of the iNOS gene in murine macrophage contains a STAT-binding gamma-activated site (GAS) [21]. STAT3 is involved in LPS-induced expression of iNOS and is partly dependent on Ser^727^ phosphorylation, which is also necessary for nuclear translocation and DNA binding [22]. Activated STAT3 also participates in the regulation of cell growth, differentiation, and survival and is essential for gp130-mediated osteoclast formation [23]. Another STAT3 target gene, MCP-1/chemokine (C–C motif) ligand 2 (Ccl2), is a chemokine belonging to the CC chemokine family that plays a critical role in the recruitment and activation of leukocytes during acute inflammation [24]. MCP-1 plays a critical role in the pathogenesis of arteriosclerosis and other vascular diseases by recruiting monocytes into the arterial wall [25]. Furthermore, MCP-1 has been implicated in cell–cell fusion of osteoclasts [26].

Capric acid, a medium chain fatty acid, is an important ingredient of coconut oil and enhances the immune system by acting as a reinforcing agent. Recently, there has been increasing evidence that deficiency of certain fatty acids may contribute to bone loss [27]. Several milk fractions and fatty acids also inhibit osteoclastogenesis in bone marrow cultures and RAW264.7 cells [28]. Fatty acids, which are merely carboxylic acids with long hydrocarbon chains, are an important source of fuel for many tissues such as heart and skeletal muscles. Based on several studies, we have extended this approach to assess the effects of capric acid in osteoclastogenesis.

In this study, we investigated the effect of capric acid on LPS-induced osteoclast differentiation in RAW264.7 cells. To understand the mechanism of capric acid, we analyzed the signal transduction pathways of NF-κB, JNK, ERK1/2, and STAT.

## Results

### Effects of capric acid on LPS-induced RAW264.7 cells

**Figure 1:**
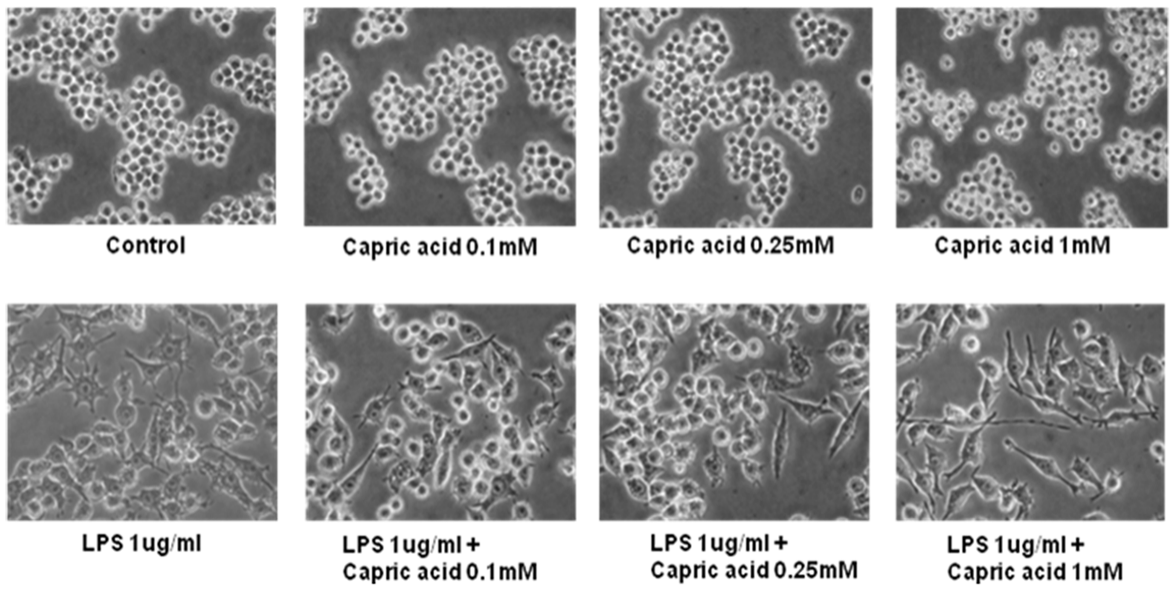
Morphological alteration of RAW264.7 cells treated with lipopolysaccharide (LPS) and capric acid. RAW264.7 cells were treated with 1 µg/ml of LPS with or without capric acid (0.1, 0.25, and 1 mM) for 24 hr.

**Figure 2:**
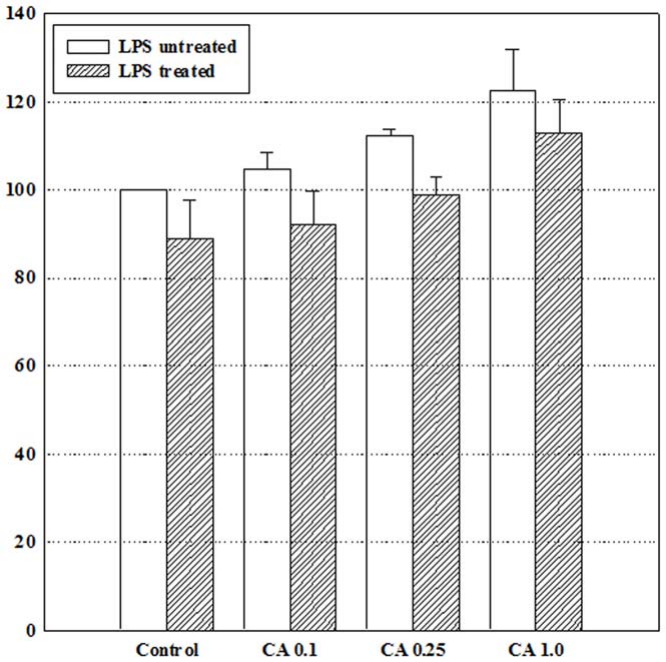
Cell viability of RAW264.7 cells treated with lipopolysaccharide (LPS) and capric acid. RAW264.7 cells were treated with or without 1 µg/ml of LPS and with capric acid (0.1, 0.25, and 1 mM) for 24 hr.

Previous studies have demonstrated that LPS induces osteoclast formation in RAW264.7 cells [12], [14], [29]. In our work, we have also used RAW264.7 cells, which can differentiate into osteoclast-like cells in the presence of LPS. To measure LPS-induced morphological changes in response to capric acid, we treated RAW264.7 cells with various concentrations of capric acid (0.1, 0.25, and 1 mM). As shown in Fig. 1, the control group and capric acid-treated groups (0.1, 0.25, and 1 mM) did not show any morphological changes, whereas the LPS-treated (1 µg /ml) group showed morphological changes. Furthermore, capric acid at 0.1, 0.25, and 1 mM induced cell proliferation by 104.9±3.6, 112.4±1.3, and 122.4±9.5% of the control (100%), respectively, and reduced the LPS-induced cytotoxic effect by 91.9±7.7, 98.6±4.3, and 113.1±7.5% of the control (100%) when compared to LPS only (89.0±8.5%), respectively (Fig. 2).

### Capric acid inhibits TRAP-positive multinucleated cell formation

**Figure 3:**
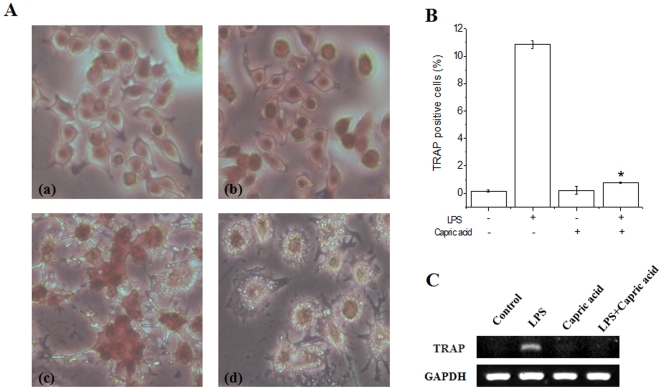
Effects of capric acid on lipopolysaccharide (LPS)-induced osteoclast formation. RAW264.7 cells were cultured with LPS in the absence or presence of capric acid for 1–3 days, and then cells were fixed and stained for tartrate resistant acid phosphatase (TRAP), respectively. (A) Multinucleated cells were observed on day 2. (a) control, (b) capric acid, (c) LPS only, (d) LPS + capric acid, (B) TRAP-positive cells were counted as osteoclasts on day 2. (C) RAW264.7 cells were cultured with LPS in the absence or presence of capric acid for 24 hr. Total RNA was isolated using Trizol reagent, and mRNA levels were determined by RT-PCR using primers for TRAP and GAPDH. Values are expressed as the mean ± SEM from triplicate cultures. *p<0.05: significantly different from the LPS-treated value.

LPS causes the formation of TRAP-positive multinucleated giant RAW264.7 cells [12]. Therefore, we examined the effects of capric acid on osteoclast differentiation in LPS-induced RAW264.7 cells using TRAP staining, a marker of osteoclasts. We confirmed LPS-induced osteoclast formation in RAW264.7 cells over 1–3 days (Fig. 3). Multinucleated cell formation was observed during the first 2 days after LPS stimulation, although there was still a small number of TRAP-positive mononuclear cells. Our results show that TRAP-positive cells were decreased significantly by capric acid (1 mM) (Fig. 3A). Moreover, LPS-stimulated TRAP mRNA (an osteoclast-specific gene) expression was also inhibited by capric acid (Fig. 3C).

### Capric acid inhibits LPS-induced NO production in RAW264.7 cells

**Figure 4:**
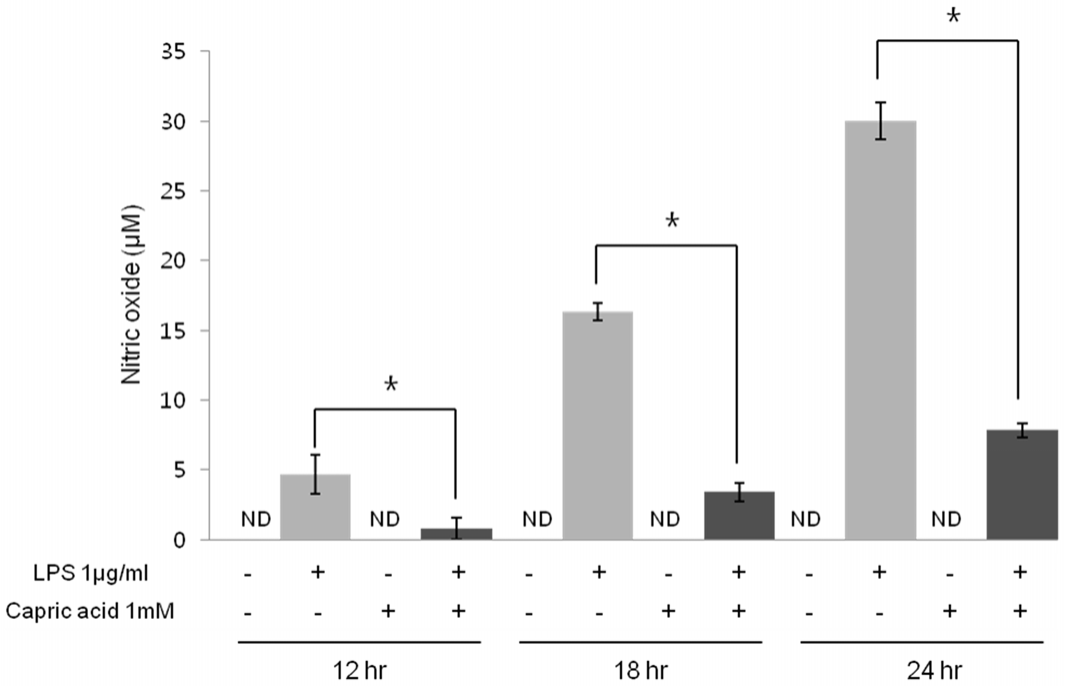
Effects of capric acid on lipopolysaccharide (LPS)-induced nitric oxide (NO) production. RAW264.7 cells were incubated with 1 µg/ml of LPS in the absence or presence of 1 mM capric acid for 12, 18, and 24 hr. The media was harvested 24 hr later and assayed for NO. Data represent the mean ± SEM for three independent experiments. *p<0.05: significantly different from the LPS-treated value.

NO is generated by iNOS and affects osteoclast formation and function. NO enhances osteoclastogenesis by mediating cell fusion [7]. To examine the effects of capric acid on LPS-induced NO production, we treated RAW264.7 cells with capric acid for 12, 18, and 24 hr. The results showed that capric acid significantly inhibited LPS-induced NO production at all tested time points (Fig. 4). Hence, our results indicate that capric acid suppressed LPS-induced osteoclast formation by inhibiting NO production.

### Capric acid inhibits LPS-induced iNOS and MCP-1 gene upregulation in RAW264.7 cells

**Figure 5:**
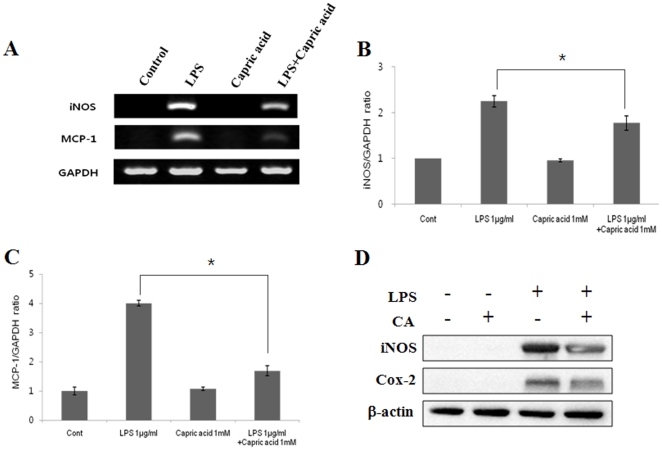
Capric acid suppresses lipopolysaccharide (LPS)-induced expression of inducible nitric oxide synthase (iNOS) and monocyte chemoattractant protein-1 (MCP-1) in RAW264.7 cells. (A) RAW264.7 cells were cultured with 1 µg/ml of LPS in the absence or presence of 1 mM capric acid for 24 hr. Total RNA was isolated using Trizol reagent, and mRNA levels were determined by RT-PCR using specific primers to iNOS, MCP-1, and GAPDH. (B, C) GAPDH gene expression levels are relative values, which were normalized. (D) RAW264.7 cells were cultured with 1 µg/ml of LPS in the absence or presence of 1 mM capric acid for 24 hr. Changes in protein expression following treatment with capric acid were measured using specific antibodies against iNOS, cyclooxygenase-2 (COX-2), and β-actin.

Because MCP-1 is reportedly involved in osteoclast cell-cell fusion and differentiation, we next examined the effects of capric acid on LPS-induced expression of iNOS and MCP-1 by RT-PCR [30]. Treatment with LPS alone markedly increased iNOS and MCP-1 gene expression, whereas treatment with capric acid significantly inhibited iNOS and MCP-1 expression (Fig. 5). iNOS and cylcooxygenase-2 (COX-2) protein expression increased following LPS treatment but decreased following capric acid treatment (Fig. 5D).

### Capric acid inhibits LPS-induced phosphorylation of Ser^727^ STAT3 in RAW264.7 cells

**Figure 6:**
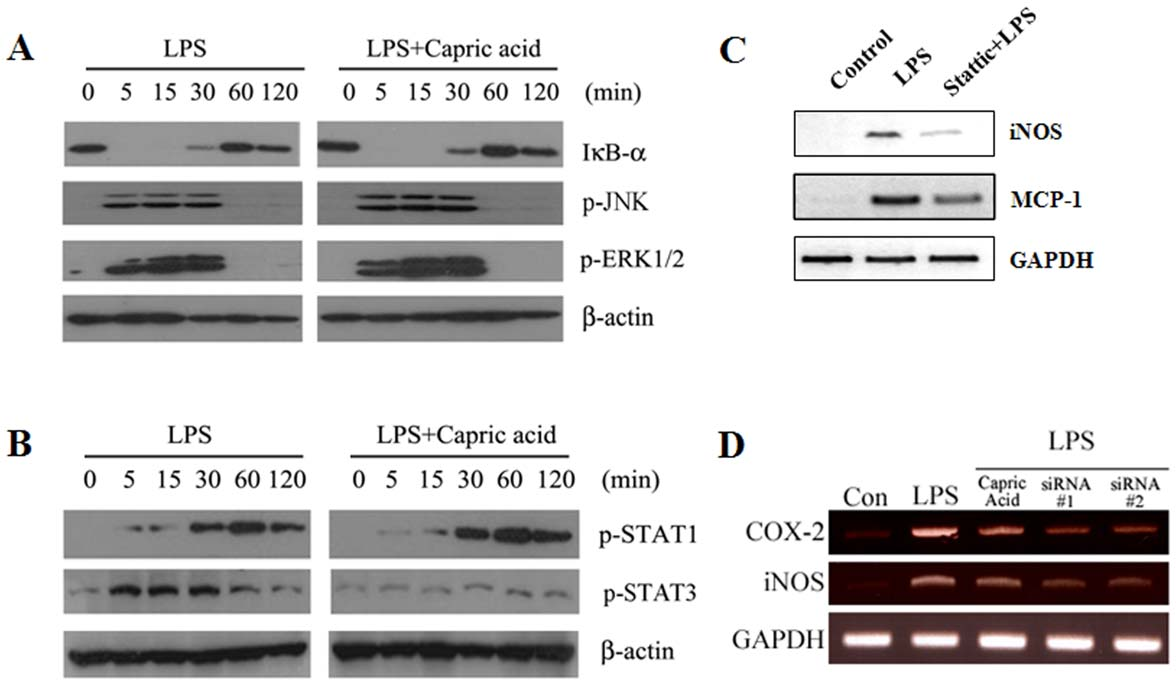
Effects of capric acid in RAW264.7 cells. (A, B) RAW264.7 cells were cultured with lipopolysaccharide (LPS) in the absence or presence of capric acid for 5, 15, 30, 60, and 120 min. The amounts of IκB-α, p-JNK, p-ERK1/2, p-STAT1, and p-STAT3 were determined by Western blot analysis. β-actin was used as an internal control. (C) RAW264.7 cells were cultured with 1 µg/ml of LPS in the absence or presence of stattic for 24 hr. Total RNA was isolated using Trizol reagent, and mRNA levels were determined by RT-PCR using primers specific to inducible nitric oxide synthase (iNOS), monocyte chemoattractant protein-1 (MCP-1), and GAPDH. All analyses were representative of at least three independent experiments. (D) Effects of capric acid or siRNAs of STAT3. STAT3 siRNA sequence #1: sense, 5′-GAA CAA CAU GUC AUU UGC UUU-3′, antisense, 5′-AGC AAA UGA CAU GUU GUU CUU-3′; STAT3 siRNA sequence #2: sense, 5′-UCA UCA UGG GCU AUA AGA UUU-3′, antisense, 5′-AUC UUA UAG CCC AUG AUG AUU-3′.

Previous studies have shown that osteoclastogenesis is promoted by activating various intracellular signaling pathways, including MAPKs, such as JNK, ERK, and P38 and transcription factors, such as NF-κB, NFATc1, and STAT [31], [32]. To clarify the molecular mechanism of capric acid during LPS-stimulated osteoclast formation, we measured transcription factor expression and phosphorylation of various signaling molecules by Western blot analysis. We treated cells with LPS for 0, 5, 15, 30, 60, and 120 min in the absence or presence of capric acid, after which activation of ERK1/2, JNK, NF-κB, STAT1, and STAT3 was determined. Capric acid had no effect on LPS-stimulated activation of NF-κB, JNK, or ERK1/2 or expression of TNF-α, IL-1β, or IL-6 (Fig. 6A and C). As shown in Fig. 6B, LPS-induced phosphorylation of STAT3 Ser^727^ was inhibited by capric acid. How capric acid inhibits LPS-induced STAT3 phosphorylation still remains to be determined. Additionally, we used stattic, an inhibitor of STAT3, to determine whether or not LPS-induced expression of iNOS and MCP-1 is regulated through STAT3 activation. The results showed that LPS-induced iNOS and MCP-1 gene expression was inhibited by stattic. Additionally, COX-2 and iNOS gene expression decreased when treated with STAT3 siRNA (Fig. 6D). These results suggest that capric acid suppressed LPS-stimulated osteoclast formation via the STAT3 pathway in RAW264.7 cells.

## Discussion

We demonstrated that capric acid inhibits LPS-induced osteoclastogenesis in RAW264.7 cells. One of the key factors in osteoclastogenesis, which is induced by osteoblasts, is activation of the RANKL receptor [33], [34]. Bone mass is controlled by the balance between the activities of osteoblasts and osteoclasts. Osteoclasts are multinuclear cells formed by cell fusion and are characterized by high TRAP expression. Recently, it was reported that LPS-induced osteoclast formation does not require RANKL and M-CSF [12]. Moreover, Hotokezaka *et al.* showed that RANKL-independent cell fusion of osteoclast-like cells can be induced by TNF-α, LPS, and peptidoglycans [14]. RAW264.7 cells act as osteoclast progenitors and differentiate into osteoclasts in response to LPS. In this study, we determined the effects of capric acid on LPS-induced osteoclast formation in RAW264.7 cells using the TRAP staining method. TRAP staining is used to stain TRAP in osteoclasts. Alkaline phosphatase and TRAP are used as markers for osteoblasts and osteoclasts, respectively. According to our results, LPS increased the formation of TRAP-positive multinucleated cells and TRAP gene expression, as shown by TRAP staining and RT-PCR. However, these events were inhibited by capric acid (Fig. 2). Therefore, we concluded that capric acid inhibits LPS-induced TRAP-positive osteoclast formation.

NO, an important multifunctional signaling molecule in bone, is produced by various cells at basal or stimulated levels. NO regulates bone formation, resorption, remodeling, mechanotransduction, and repair under physiological or pathophysiological conditions. NO produced endogenously or supplied by NO donors exerts potent biphasic actions that profoundly affect the recruitment, proliferation, differentiation, activity, and/or survival of osteoclasts and osteoblasts, their precursors, and other cells within the bone [35], [36], [37]. Low levels of NO may support osteoblast bone formation and osteoclast-mediated bone remodeling (both basal and cytokine-induced) [38], [39], whereas high NO levels and NO-generating compounds inhibit osteoclast formation and bone resorption and prevent bone loss during severe inflammation or in estrogen-deficient animals [40], [41], [42]. Here, we report that capric acid inhibited NO production and iNOS mRNA expression (Figs. 3 and 4). Therefore, our results showed that LPS-induced NO production is inhibited by capric acid, whereas NO may act as a mediator of osteoclast formation.

Next, we considered which signaling molecules affect LPS-induced NO production and iNOS expression. There are a large number of binding sites for transcription factors in the iNOS region, including NF-κB, AP1, STATs, and CCAAT/enhancer-binding protein (C/EBP), based on cell type and stimulus [43]. Among the transcription regulators in the promoter regions of iNOS, NF-κB seems to be essential for LPS-induced inflammatory cytokine production [44]. An abundant amount of pro-inflammatory cytokines at sites of inflammation promotes osteoclast differentiation and activation. However, our results showed no effect of capric acid on the NF-κB pathway, consistent with the finding that capric acid had no effect on LPS-induced expression of the pro-inflammatory cytokines IL-1β, TNF-α, or IL-6 (Fig. 5B and C).

STAT is another key signaling molecule involved in the cytokine-induced inflammatory response [45]. Among the STAT family, STAT3 is an important transcription factor for regulating iNOS gene expression, and STAT3 DNA binding is affected by phosphorylation of Ser 727 or/and Tyr 705 [46]. Previous studies have shown that a STAT-binding GAS (interferon-gamma activated sequence) is necessary for iNOS expression in LPS-induced RAW264.7 cells [47]. Furthermore, STAT3 mutant mice exhibit decreased bone density, bone volume, and an increased number of TRAP-positive osteoclasts [48]. These results suggest that STAT3 plays a negative role in the regulation of osteclastogenesis. Our present study found that treatment with LPS alone markedly increased STAT3 phosphorylation at Ser 727, whereas phosphorylation was decreased strongly by capric acid (Fig. 5B). As shown in Fig. 4, capric acid inhibited iNOS and MCP-1 gene expression (STAT3 target genes). Additionally, LPS-induced iNOS and MCP-1 mRNA expression was inhibited by the STAT3 inhibitor stattic (Fig. 5D). Based on these findings, we concluded that capric acid exerts a potent inhibitory effect by inhibiting iNOS expression and by activating STAT3 (Ser 727) in LPS-treated RAW264.7 cells.

In conclusion, we demonstrated the inhibitory effects of capric acid on LPS-induced osteoclastogenesis by targeting NO production via the STAT3 pathway. Fatty acids are important components of a normal diet, and the manipulation of dietary fatty acid composition may influence bone resorption, bone formation, and bone mass. Further elucidation of the mechanism of capric acid regulation should contribute to the discovery of novel therapeutic approaches for treating various types of inflammatory bone destruction.

## Materials and Methods

### Cell culture

RAW264.7, a mouse macrophage cell line, was obtained from the ATCC (Rockville, MD, USA). Cells were cultured in 10 mm plates and maintained in high glucose Dulbecco's Modified Eagle Medium (DMEM) supplemented with 2 mM glutamine, antibiotics (1% penicillin/streptomycin), and 10% heat-inactivated fetal bovine serum (FBS) in a 37°C humidified incubator containing 5% CO_2_.

### Reagents

LPS, capric acid, L-NMMA, and a TRAP kit were purchased from Sigma-Aldrich (St. Louis, MO, USA). Antibodies specific to p-JNK, JNK, p-ERK, and ERK along with horseradish peroxidase (HRP)-conjugated rabbit and mouse IgG antibodies were purchased from Cell Signaling Technology (Danvers, MA, USA). Antibodies to IκB-α were purchased from Santa Cruz Biotechnology (Santa Cruz, CA, USA). An NO detection kit was purchased from iNtRON Biotech (Seoul, Korea). An RNeasy mini kit was purchased from Qiagen Inc. (Valencia, CA, USA).

### TRAP staining

RAW264.7 cells were plated at a density of 5×10^5^ cells/well in 24-well plates for 12 hr and then treated with the indicated compounds for an additional 24, 48, and 72 hr. The supernatant was removed, and the cells were washed twice with PBS. Fixation solution was added to the cells and then removed with PBS. TRAP staining solution (50 mM acetate buffer, 30 mM sodium tartrate, 0.1 mg/ml of Naphtol AS-MX phosphate, 0.1% w/v Triton X-100, and 0.3 mg/ml of Fast Red Violet LB stain) was added to the cells for 1 hr. The solution was then removed, and the cells were washed twice with PBS. Cell morphology was detected by microscopic observation.

### 3-[4,5-dimetylthiazol-2-yl]-2,5-diphenyltetrazolium bromide (MTT) assay

Cell viability was determined using a colorimetric 3-[4,5-dimetylthiazol-2-yl]-2,5-diphenyltetrazolium bromide (MTT) assay. RAW264.7 cells (5×10^4^ cells/well) were cultured in 96-well plates for 24, 48, and 72 hr after LPS treatment with or without capric acid. MTT solution (20 µl; 5 mg/ml) was added, and the cells were incubated at 37°C for an additional 4 hr. After washing out the supernatant, the insoluble formazan product was dissolved in DMSO. Then, the optical density of the 96-well culture plates was measured using an ELISA reader at 570 nm. The optical density of formazan formed in the untreated control cells was considered 100% viability.

### NO analysis

RAW264.7 cells were plated at a density of 5×10^4^ cells/well in 96-well plates for 12 hr and then treated with the indicated compounds for an additional 12, 18, and 24 hr. The supernatant from the cultured cells was centrifuged to remove cell debris and transferred to 96-well plates. The supernatant was then reacted using a nitric oxide detection kit (iNtRON Biotech). Values were calculated by measuring the absorbance at 540 nm using a plate reader.

### RT-PCR

RAW264.7 cells were treated with each of the compounds (1 µg/ml LPS and 1 mM capric acid) for 24, 48, and 72 hr, followed by washing with PBS. Total RNA was isolated with an RNeasy Mini kit (Qiagen), and the total RNA concentration was detected using a spectrophotometer. Total RNA (1 µg) was converted to cDNA with cDNA Synthesis Master Mix (GenDEPOT; Barker, TX, USA). PCR was performed using Maxim PCR Premix (iNtRON Biotech). The PCR primers were as follows: TRAP- F; 5′-TCC CCT GGT ATG TGC TGG-3′ and R; 5′-GCA TTT TGG GCT GCT GA-3′, iNOS- F; 5′-GCA GAA TGT GAC CAT CAT GG-3′ and R; 5′-ACA ACC TTG GTG TTG AAG GC-3′, MCP-1- F; 5′-GAA GGA ATG GGT CCA GAC AT-3′ and R; 5′-ACG GGT CAA CTT CAC ATT CA-3′, COX-2- F; 5′-CAC TAC ATC CTG ACC CAC TT-3′ and R; 5′-ATG CTC CTG CTT GAG TAT GT-3′, STAT3- F; 5′-AGA ACC TCC AGG ACG ACT TTG-3′ and R; 5′-TCA CAA TGC TTC TCC GCA TCT-3′, GAPDH-F; 5′-CAT GAC CAC AGT CCA TGC CAT CAC T-3′ and R; 5′-TGA GGT CCA CCA CCC TGT TGC TGT A-3′, The amplification sequence protocol was conducted at 95°C for 30 s, 58°C for 30 s, and 72° for 45 s for each cycle. PCR products were separated by electrophoresis on 1% agarose gels and visualized with ethidium bromide staining.

### Western blot analysis

RAW264.7 cells were lysed in protein extraction solution (iNtRON Biotech). The lysate was centrifuged at 13,000 rpm for 20 min at 4°C to remove cellular debris. Protein concentrations of the extracts were determined by the Bradford assay (Bio-Rad Laboratories; Hercules, CA, USA). Equal amounts of protein were separated by SDS polyacrylamide gel electrophoresis, transferred to a nitrocellulose membrane, and blocked with 5% skim milk in TBST for 1 hr. The membranes were then incubated with various primary antibodies, which were diluted with TBST at 4°C with gentle shaking overnight. After washing, the membrane was incubated with HRP-goat anti-mouse IgG (H + L) conjugate antibody (Zymed Laboratories; San Francisco, CA, USA) diluted in TBST (1∶2,000) for 1 hr. After washing several times with TBST, the blots were visualized with enhanced chemiluminescence reagents (Amersham Biosciences; Chalfont, Buckinghamshire, UK).

### siRNA transfection

siRNAs targeting STAT3 mRNA were designed and purchased from Genolution Pharmaceuticals (Seoul, Korea). The TransIT-TKO® reagent from Mirus Bio (Madison, WI, USA) was used for siRNA delivery. RAW264.7 cells were seeded at 3×10^5^ cells/well in 6-well plates containing 2.5 ml DMEM and 10% FBS for 12 hr. DMEM (250 µl) containing 10 nM of siRNA and 7.5 µl of TransIT-TKO® reagent were incubated at room temperature for 30 min and added to each well. After a transfection with STAT3 siRNA for 24 hr, the medium was replaced with normal medium and used for the next experiments.

### Statistical analysis

Statistical analyses were performed using SPSS (Statistical Package for the Social Sciences) ver. 12.0 software (SPSS, Inc., Chicago, IL, USA). Each datum represents the mean ± SEM for different experiments under the same conditions. Statistical significance was compared between each treated group and the control using the independent *t*- test. *P* values <0.05 were considered statistically significant.
